# Social brain dysfunctions in patients with Parkinson’s disease: a review of theory of mind studies

**DOI:** 10.1186/2047-9158-2-7

**Published:** 2013-03-28

**Authors:** Rwei-Ling Yu, Ruey-Meei Wu

**Affiliations:** 1 Department of Psychology, National Taiwan University, Taipei, Taiwan; 2 Department of Neurology, National Taiwan University Hospital, College of Medicine, National Taiwan University, 10002, Taipei, Taiwan

**Keywords:** Theory of mind, Social cognition, Neuropsychology, Cognitive function, Parkinson’s disease

## Abstract

Human social interaction is essential in daily life and crucial for a promising life, especially in people who suffer from disease. Theory of Mind (ToM) is fundamental in social interaction and is described as the ability to impute the mental states of others in social situations. Studies have proposed that a complex neuroanatomical network that includes the frontal cortex mediates ToM. The primary neuropathology of Parkinson’s disease (PD) involves the frontal-striatal system; therefore, patients with PD are expected to exhibit deficits in ToM. In this review, we summarize the current research with a particular focus on the patterns of impaired ToM, potential mediators of ToM, and the impact of ToM deficits on clinical disability in PD. Further studies to investigate the progression of ToM and its relationship with dementia in subjects in PD are needed.

## 

Parkinson’s disease (PD) is a progressive neurodegenerative disorder pathologically characterized by the selective loss of dopaminergic neurons in the substantia nigra. Clinical manifestations include resting tremors, rigidity, bradykinesia, and postural instability. Besides motor impairment, dopamine depletion can also affect cognitive and affective behavior [1]. Indeed, there is some evidence to suggest that PD can develop years before the hallmark of motor features [2]. Increasing evidence shows that patients with PD have non-motor symptoms, such as mild cognitive impairment [3-12], dementia [13,14], and olfactory dysfunction [15]. Of note, it has been suggested that Theory of Mind (ToM), a concept within social cognitive neuroscience that refers to the ability to attribute mental state to oneself and others, is impaired in PD patients. In fact, it has been demonstrated that some PD patients possess social interaction impairment, such as being unable to detect or understand a caregiver’s emotion and making inappropriate responses in a social situation. Furthermore, our latest study provided first direct evidence in Taiwanese population that non-demented PD patients exhibit ToM dysfunction early in the disease process. Hereby, we review the literature regarding the evidence for ToM impairment and its impact in patients with PD; the potential mediators related to ToM deficit.

## Introduction

ToM is a complex neuropsychological concept that refers to the ability to infer another’s thoughts, intentions, or emotions to oneself. The ability to interact effectively in a social situation is a necessary skill for successful communication. A key component of ToM is the ability to recognize the mental state of others. Premack and Woodruff first coined the term “ToM” in a paper reporting on the mental states of chimpanzees [16]. Since then, the ToM construct has been employed in many human studies, especially those investigating psychiatric disorders [17] and development disorders, such as autistic spectrum disorders [18].

Whether or not ToM abilities improve or deteriorate with age has also received attention. Given the well-documented age-related functional decline of the frontal lobes, an area of the brain implicated in ToM, it seems logical that ToM abilities should also deteriorate. However, related research findings have been controversial. For example, some scholars have suggested that performances on ToM tests declines significantly in people older than 55 y/o [19-23]. In contrast, other studies reported that elderly individuals demonstrate preserved or superior ToM abilities due to their well-seasoned life [24,25]. The idea that an age-related decline in social understanding has been proposed to be partially mediated by declining fluid intelligence [26].

From normal ageing, research migrated to exploring ToM in neurodegenerative disease, such as fronto-temporal dementia [27], Alzheimer’s dementia [28,29], and various basal ganglia disorders, including PD [30]. Given the importance of ToM in social communication and life of quality in PD, we reviewed the status of the research in ToM including the clinical manifestation of cognition, health-related quality of life, effect of pharmacological and surgical therapy and the relationship of ToM with non-motor symptoms of PD. The future direction is also discussed at the end particularly on the progression of ToM and dementia in PD.

## The search strategy and the selection criteria

A comprehensive review of the literature was conducted through PubMed and PsychINFO databases using the following keywords: “Parkinson’s disease” and “Theory of mind.” A literature search was undertaken for articles published between January 2000 and December 2012. The studies considered eligible were empirical studies written in English and published in peer-reviewed journals. The search retrieved 17 original researches and abstracts were further scrutinized to include only those reports that through the study filter. The study inclusion criteria were: (1) studies provide the quantitative ToM data and focused in patients with PD and (2) comparison of PD with healthy controls (HCs). Exclusion criteria were: (1) the study topic is not ToM; and (2) studies focused on ToM in other population and with no relevant data about ToM in PD. Finally, 13 articles [31-43] were included in this review.

## ToM dysfunction is common in PD

Few studies focusing on ToM and PD were carried out at the beginning of the 21st century. The first study, conducted by Saltzman et al. [31], compared the performance of 11 non-demented PD patients [age: 71 years old, Hoehn and Yahr stage, H&Y: 2.5, MMSE >26] with 8 age-matched HCs [31]. They demonstrated that PD patients performed worse in false-belief stories and during a spy model task; specifically, PD patients were less able to make correct predictions based on inferences regarding a story-character’s belief, and they had more difficulty planning a course of action that would deceive another person. Another study, by Mengelberg and colleagues (2003), reported that non-demented PD patients demonstrated deficits in false beliefs, short-passage tasks, and first-order story tasks, while their performance of second-order story tasks was compble to controls. Notably, the authors found more pronounced ToM deficits in the PD group with higher depression scores, and thus suggested that depression impacts on ToM processing [32].

Over the past 6 years, studies have increasingly begun to explore ToM ability in non-demented PD patients. Taken together, the findings of these studies suggest that ToM deficits occur during the pathological course of PD and can represent additional non-motor symptoms. It is believed that ToM has 2 subcomponents: cognitive (”cold”) and affective (“hot”) [44]. Cognitive ToM refers to the ability to make inferences about another’s cognitive state, such as their intentions and motivations, while affective ToM refers to the ability to recognize another’s affective state, such as their feelings and emotions [45]. Some psychological tests, such as the Faux Pas Recognition task (FPR) and the Reading the Mind in the Eyes test (REMT), were developed to measure ToM ability. The FPR is a verbal ToM task, which reflects an individual’s ability to detect and attribute inappropriate behavior in a social situation. The latter is a visual ToM task that requires individuals to recognize or describe feelings. An example question would be, “which of the following adjectives best describes the eye region shown: excited, relieved, shy, or despondent”.

Bodden et al. examined affective ToM in 21 PD patients (age: 63.7, duration: 5.1 years, H&Y: 2.5, MMSE: 29) and 21 HCs using REMT; compared to the control subjects, PD patients scored lower on the REMT. Other researchers found similar results and suggested that PD patients could not accurately detect the feelings of another person by simply looking at their eye region, even in the initial [41] and early stages [39,41] of the disease. However, this finding could not be replicated by other researchers [33,35,37,38], who instead reported that PD patients may have preserved affective ToM performances in the early stage of the disease [33,35,37,38].

These controversial results might be due to methodological differences employed; for example, the REMT version used in the study by Roca et al. is an abbreviated version in which 15 stimuli were used instead of the usual 36, and the patients were asked to choose between 2 instead of 4 adjectives. Furthermore, the sample size in the studies were quite small (n= 21 vs. 17) [33,35]. The patients selected in the studies by Peron et al. [35,37] were relatively young (average age: 56 and 53 years old). Pondering the disease duration (10.2 vs. 10.5 years), these patients are considered to be young onset Parkinsonism rather than late onset idiopathic PD.

Researchers have found that PD patients have impaired cognitive ToM. This is identified using various tasks, such as the first-order false-belief story [31,32], the second-order false-belief story [34,36], and the cognitive components of the FPR test [35,38] and the Yoni test [36]. Many studies used FPR to assess cognitive ToM; they found that PD patients have more difficulties detecting the faux pas in a social situation and have declined ability in recognizing the reason why a person in the story made an inappropriate remark [35,38]. Roca and colleagues (2010) compared 36 early stage PD patients [16 medicated (age: 63.4, duration: 1.69 years, H&Y: 1.42, MMSE: 29) and 20 drug free (age: 63.5, duration: 1.23 years, H&Y: 1.33, MMSE: 28.26)] and 35 HCs [38]; they found cognitive ToM impairment in all patients regardless of the presence or absence of dopamine replacement therapy (DRT). Nevertheless, Peron et al. [35] reported that only advanced PD patients [n= 27, age: 56.6 years old, duration: 10.2 years, H&Y: 1.3(on state) and 2.5(off), MDRS: 139.1] performed poorly on the intention attribution question of the FPR. Moreover, Roca et al. [41] and Yu et al. [43] suggested that not all early non-demented PD patients have impaired cognitive ToM. Both their studies revealed that PD patients performed similarly to their intelligence-matched HCs; in this case, intelligence was measured by the verbal intelligence quotient (VIQ), and the Raven’s Colored Progressive Matrices (RCPM). Recently, Costa et al. [40] found that only patients with executive dysfunction have impaired cognitive ToM. Thus, possible confounders, such as intelligence, executive function, and the heterogeneity of PD should be taken into account in future studies.

## How ToM affect PD patients in daily living?

Studies have explored the impact of cognitive and affective ToM dysfunction on real life especially the quality of life (QoL) [36,42]. Both studies used the PD questionnaire (PDQ-39) to measure the health-related QoL. Bodden et al. [30] first found a significant correlation between total PDQ-39 score and measures of affective but not cognitive ToM among patients with advanced stage PD (H&Y: 2.5, range: I to III). Subsequently, Santangelo and colleagues (2012) excluded depressed PD patients and included early stage patients (H&Y: 1.5, range: I to II) in order to explore this issue, and their results showed that cognitive ToM was associated with 2 domains, social support and cognitive deficits subscales, of PDQ-39. Therefore, it was suggested that defects in the cognitive aspect of ToM might have a more negative impact on QoL compared to the affective component. Taken together, these findings imply that ToM plays an important role in daily living and affects QoL. However, more research is still needed to explore the impact of ToM on real life and social interactions.

## What factors affecting ToM in PD ?

The literature to date suggests that the degree of ToM alteration may vary in PD patients, depending on many factors in disease course, including the clinical characteristics, non-motor symptoms, and clinical management.

### ToM descending as the disease progression

Summarizing all of the literature cited in this review, the majority of studies report that PD patients in the early stages of the disease progression may have preserved affective ToM performances [33,35,37,38], even 10 years after disease onset [35], with mild difficulties sometimes emerging after 5 years [39]. However, impairment of cognitive component in the ToM could be observed in both the early [35,38,42] and moderate stage [31,32,34-36] of the disease. It is believed that different areas of the frontal cortices may be responsible for differential ToM processes. There is some evidence to suggest that the ventromedial prefrontal regions are crucial for processing affective ToM [46,47], while the dorsolateral prefrontal ones have been implicated in cognitive ToM [48,49]. Recently, it has been revealed that cognitive and affective subcomponents of ToM are linked to different frontostriatal circuitries [30], which are known to be affected by PD [50].

**Figure 1:**
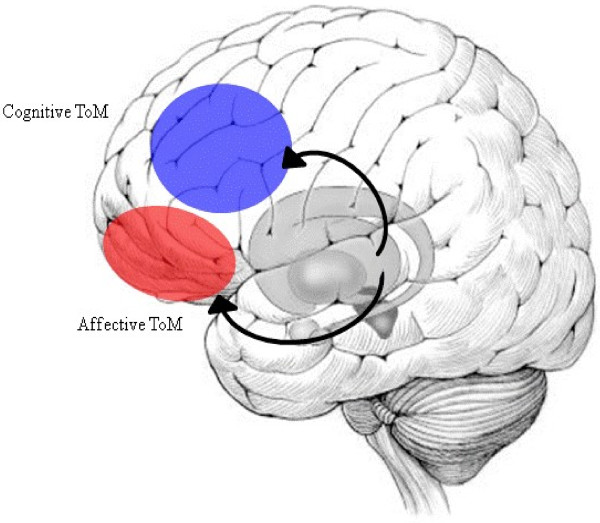
**The model of ToM processing in PD.** Blue: Dorsolateral prefrontal cortex, Red: Orbitofrontal cortex.

Here, we figured the current model of ToM processing in PD (Figure 1). Affective ToM is believed to be predominately mediated by the orbital frontostriatal (OFS) circuit, while cognitive ToM might additionally be related to the dorsolateral frontostriatal (DLFS) loop [30,51]. During the early stages of PD, dopamine is mostly depleted from the dorsolateral head of the caudate nucleus, an area involved in the DLFS circuit, whereas the processes based on the OFS circuit are almost completely preserved. As PD progresses towards the advanced stage, in which the prefrontal cortex is directly affected by the neuropathology of PD, dopamine depletion within the striatum also affects the OFS circuit, thus impairing related functions.

### The role played by other non-motor symptoms in ToM processing

Several studies have showed that neuropsychological function, especially the frontal-related function, may be a potential factor related to ToM. With regard to neuropsychiatric symptoms, Mengelberg and Siegert (2003) first mentioned that emotion disturbance might influence ToM processing in PD; thus, the following researchers excluded PD patients with neuropsychiatric symptoms in their studies. Recently, Santangelo and colleagues (2012) investigated the relationship between neuropsychiatric symptoms and ToM. They found that affective ToM dysfunction is related to severity of apathy, as assessed by the Apathy Evaluation Scale, and behavioral disturbances, as assessed by the frontal behavioral inventory. The findings of this study highlight the need for future research to investigate the contribution of ToM to behavioral disturbances, such as pnoia and delusions, which are common in dementia.

Regarding the cognitive function, most studies have focused on executive function (EFs), which traditionally is closely related to the frontal lobe and vulnerable cognitive function in PD. Until now, the relationship between EFs and ToM remained subject to debate. Some scholars have demonstrated double dissociations between ToM and EFs by controlling for EFs in their results [28,52-54]; while others have claimed that ToM is dependent upon EFs [55-57]. Although controversial results exist, based on correlation analysis, studies suggest that ToM ability is related to EFs, such as mental shifting [42,43], concept formation [43], reasoning [36], and inhibition abilities [35]. Recently, Costa et al., [40] directly compared PD patients with and without intact EFs and found that PD patients with intact EFs have reduced ability to perform ToM tasks. This association can be explained by the role played by EFs, which are capable of compensating for the deficits of ToM. Therefore, PD patients might generate advanced ToM by utilizing their EFs. EFs is a complex construct that includes people’s abilities to plan, manage, organize information, initiate and inhibit behaviors, and think abstractly and flexibly. Hence, future work should investigate this function in a more comprehensive manner to determine the specific role of EF, especially with regard to the roles of inhibitory control and working memory in the emergence and expression of ToM in social situation.

The majority of participants in studies cited here were non-demented PD patients. In these studies, the deficits precede the development of dementia as defined by the MMSE or MDRS, which have less sensitivity to detect the PDD [7,58]. It has been suggested that a specturm may exist from intact mental status to dementia. Indeed, the transitional state, social function is a key point that differeniates dementia from mild cognitive impairment (MCI) [59,60]. So far, no empirical research has investigated this related issue. With regard to the other disease of the spectrum of Lewy body disease, Dementia with Lewy bodies (DLB), by the progression of pathology in Lewy body deposition or tau protein, the frontal lobe regions implicated in ToM are affected by these abnormal inclusion bodies [61]. Thus, ToM could be regarded as the same pathological degenerative component that predicts the presence of dementia in PD. Much remains to be done, studies should be undertaken to determine whether ToM ability is altered in PD patients with MCI or dementia in the hope of identifying whether ToM is a decisive factor in the development of PDD.

### The influence of pharmacological therapy and Deep Brain Stimulation (DBS) in ToM processing

Even if it is acknowledged that the dopaminergic system is involved in areas critical for intact ToM, the exact relationship between dopamine and ToM has not yet been fully established [35,38]. Peron et al. [35] explored the involvement of the dopaminergic pathways in ToM by comparing ToM performances of early stage PD patients with and without DRT; they found no difference between medicated and unmedicated PD patients and suggested that dopaminergic pathways are not involved in ToM. Later, Roca et al. [38] administered ToM tasks to both medicated and de novo PD patients and also found no significant differences between the 2 groups; in addition, no significant correlations were revealed between ToM performance and the levodopa equivalent daily dose within the medicated group. It is difficult to draw a conclusion from these preliminary data because of methodological limitations, such as the learning effect of tasks, the small sample size, and the indirect testing of the dopaminergic pathways. These findings may also represent that ToM deficits not be attributed to dopaminergic depletion alone, other deficits of neurotransmitters, such as serotonin or norepinephrine might also influence the ToM processing.

DBS is a surgical treatment that inactivates parts of the brain that cause PD. It has provided remarkable therapeutic benefits for otherwise treatment-resistant movement disorders. Only Peron and colleagues (2010) investigated the effect of DBS in the subthalamic nucleus (STN) on ToM of PD patients [37]. They conducted ^18^FDG-PET scans in 13 PD patients [age: 53.3 years old, duration: 10.5 years, H&Y: 1.9 (pre-DBS) and 1.2 (post-DBS), MDRS: 141.4 (pre-DBS) and 141.1 (post-DBS), levodopa dosage: 1081.1 (pre-DBS) and 625.8 (post-DBS)] in pre- and post-STN DBS conditions and correlated changes in their glucose metabolism with modified performances on the REMT. Regarding the affective ToM, the authors found postoperative PD performances were worse than HCs and preoperative PD performances, whereas there was no difference between preoperative PD and HCs performances. The authors suggested that STN DBS impairs ToM because of the overlap between the limbic system, which is modulated by STN DBS in PD, and the brain network that mediates ToM. Yet, further research is needed to explore the influence of DBS in ToM by employing an exhaustive task, which covers both cognitive and affective components of ToM.

## Conclusion and future directions

The conclusions of our review are as such: (1) ToM deficits can be detected in PD patients without dementia and even at the time of PD diagnosis, (2) ToM abilities are associated with reduced health-related QoL, (3) clinical and other non-motor symptoms are associated with ToM processing in PD patients.

The aforementioned preliminary research data sketches out the nature of ToM in PD. About one-third of PD patients develop dementia during the course of the disease and the presence of dementia impacts greatly on patients and caregivers. To our knowledge, no studies have investigated whether ToM is a biomarker of PDD. Further studies are needed to investigate whether ToM deficits in PD are linear, whether different subcomponents deficits have a different evolution, and the prognostic utility of ToM in the development of dementia.

## Competing interests

The authors declare that they have no competing interests.

## Authors’ contributions

RLY and RMW contributed to the execution, critique and revision of the manuscript. Both authors read and approved the final manuscript.

## References

[B1] Alexander GE DeLong MR Strick PL pllel organization of functionally segregated circuits linking basal ganglia and cortex Annu Rev Neurosci 1986 9 357 381 10.1146/annurev.ne.09.030186.002041 3085570

[B2] Haas BR Stewart TH Zhang J Premotor biomarkers for Parkinson’s disease - a promising direction of research Transl Neurodegener 2012 1 11 10.1186/2047-9158-1-11 23211054 PMC3514104

[B3] Yu RL Wu RM Tai CH Lin CH Hua MS Feeling-of-knowing in episodic memory in patients with Parkinson's disease with various motor symptoms Mov Disord 2010 25 1034 1039 10.1002/mds.23017 20131392

[B4] Aarsland D Bronnick K Larsen JP Tysnes OB Alves G Cognitive impairment in incident, untreated Parkinson disease: the Norwegian ParkWest study Neurology 2009 72 1121 1126 10.1212/01.wnl.0000338632.00552.cb 19020293

[B5] Poletti M Frosini D Pagni C Baldacci F Nicoletti V Tognoni G Lucetti C Del Dotto P Ceravolo R Bonuccelli U Mild cognitive impairment and cognitive-motor relationships in newly diagnosed drug-naive patients with Parkinson's disease J Neurol Neurosurg Psychiatry 2012 83 601 606 10.1136/jnnp-2011-301874 22492216

[B6] Aarsland D Bronnick K Williams-Gray C Weintraub D Marder K Kulisevsky J Burn D Barone P Pagonabarraga J Allcock L Mild cognitive impairment in Parkinson disease: a multicenter pooled analysis Neurology 2010 75 1062 1069 10.1212/WNL.0b013e3181f39d0e 20855849 PMC2942065

[B7] Mamikonyan E Moberg PJ Siderowf A Duda JE Have TT Hurtig HI Stern MB Weintraub D Mild cognitive impairment is common in Parkinson’s disease patients with normal Mini-Mental State Examination (MMSE) scores Parkinsonism Relat Disord 2009 15 226 231 10.1016/j.parkreldis.2008.05.006 18595765 PMC2668811

[B8] Sollinger AB Goldstein FC Lah JJ Levey AI Factor SA Mild cognitive impairment in Parkinson's disease: subtypes and motor characteristics Parkinsonism Relat Disord 2010 16 177 180 10.1016/j.parkreldis.2009.11.002 19939721 PMC3622717

[B9] Yu RL Wu RM Tai CH Lin CH Cheng TW Hua MS Neuropsychological profile in patients with early stage of Parkinson’s disease in Taiwan Parkinsonism Relat Disord 2012 18 1067 1072 10.1016/j.parkreldis.2012.06.002 22749792

[B10] Litvan I Aarsland D Adler CH Goldman JG Kulisevsky J Mollenhauer B Rodriguez-Oroz MC Troster AI Weintraub D MDS Task Force on mild cognitive impairment in Parkinson's disease: critical review of PD-MCI Mov Disord 2011 26 1814 1824 10.1002/mds.23823 21661055 PMC3181006

[B11] Caviness JN Driver-Dunckley E Connor DJ Sabbagh MN Hentz JG Noble B Evidente VG Shill HA Adler CH Defining mild cognitive impairment in Parkinson's disease Mov Disord 2007 22 1272 1277 10.1002/mds.21453 17415797

[B12] Janvin CC Larsen JP Aarsland D Hugdahl K Subtypes of mild cognitive impairment in Parkinson's disease: progression to dementia Mov Disord 2006 21 1343 1349 10.1002/mds.20974 16721732

[B13] Nussbaum M Treves TA Inzelberg R Rabey JM Korczyn AD Survival in Parkinson's disease: the effect of dementia Parkinsonism Relat Disord 1998 4 179 181 10.1016/S1353-8020(98)00039-X 18591108

[B14] Reichmann H Schneider C Lohle M Non-motor features of Parkinson’s disease: depression and dementia Parkinsonism Relat Disord 2009 15 Suppl 3 S87 S92 20083017 10.1016/S1353-8020(09)70789-8

[B15] Chen W Tan YY Hu YY Zhan WW Wu L Lou Y Wang X Zhou Y Huang P Gao Y Combination of olfactory test and substantia nigra transcranial sonography in the differential diagnosis of Parkinson’s disease: a pilot study from China Transl Neurodegener 2012 1 25 10.1186/2047-9158-1-25 23267690 PMC3582583

[B16] Premack D Woodruff G Does the chimpanzee have a theory of mind? Behavioral and Brain Sciences 1978 1 515 526 10.1017/S0140525X00076512

[B17] Bora E Yucel M Pantelis C Theory of mind impairment: a distinct trait-marker for schizophrenia spectrum disorders and bipolar disorder? Acta Psychiatr Scand 2009 120 253 264 10.1111/j.1600-0447.2009.01414.x 19489747

[B18] Baron-Cohen S Tager-Flusberg H Cohen DJ Understanding other minds: perspectives from developmental cognitive neuroscience 2000 2 Oxford: Oxford University Press

[B19] Maylor EA Moulson JM Muncer AM Taylor LA Does performance on theory of mind tasks decline in old age? Br J Psychol 2002 93 465 485 10.1348/000712602761381358 12519529

[B20] Duval C Piolino P Bejanin A Eustache F Desgranges B Age effects on different components of theory of mind Conscious Cogn 2011 20 627 642 10.1016/j.concog.2010.10.025 21111637

[B21] Phillips LH MacLean RD Allen R Age and the understanding of emotions: neuropsychological and sociocognitive perspectives J Gerontol B Psychol Sci Soc Sci 2002 57 P526 P530 10.1093/geronb/57.6.P526 12426435

[B22] Slessor G Phillips LH Bull R Exploring the specificity of age-related differences in theory of mind tasks Psychol Aging 2007 22 639 643 17874961 10.1037/0882-7974.22.3.639

[B23] Pardini M Nichelli PF Age-related decline in mentalizing skills across adult life span Exp Aging Res 2009 35 98 106 10.1080/03610730802545259 19173104

[B24] Happe FG Winner E Brownell H The getting of wisdom: theory of mind in old age Dev Psychol 1998 34 358 362 9541787 10.1037//0012-1649.34.2.358

[B25] Charlton RA Barrick TR Markus HS Morris RG Theory of mind associations with other cognitive functions and brain imaging in normal aging Psychol Aging 2009 24 338 348 19485652 10.1037/a0015225

[B26] Sullivan S Ruffman T Social understanding: How does it fare with advancing years? Br J Psychol 2004 95 1 18 10.1348/000712604322779424 15005864

[B27] Kipps CM Hodges JR Theory of mind in frontotemporal dementia Soc Neurosci 2006 1 235 244 10.1080/17470910600989847 18633790

[B28] Gregory C Lough S Stone V Erzinclioglu S Martin L Baron-Cohen S Hodges JR Theory of mind in patients with frontal variant frontotemporal dementia and Alzheimer’s disease: theoretical and practical implications Brain 2002 125 752 764 10.1093/brain/awf079 11912109

[B29] Poletti M Enrici I Adenzato M Cognitive and affective Theory of Mind in neurodegenerative diseases: neuropsychological, neuroanatomical and neurochemical levels Neurosci Biobehav Rev 2012 36 2147 2164 10.1016/j.neubiorev.2012.07.004 22819986

[B30] Bodden ME Dodel R Kalbe E Theory of mind in Parkinson's disease and related basal ganglia disorders: a systematic review Mov Disord 2010 25 13 27 10.1002/mds.22818 19908307

[B31] Saltzman J Strauss E Hunter M Archibald S Theory of mind and executive functions in normal human aging and Parkinson’s disease J Int Neuropsychol Soc 2000 6 781 788 10.1017/S1355617700677056 11105468

[B32] Mengelberg A Siegert RJ Is theory-of-mind impaired in Parkinson’s disease? Cogn Neuropsychiatry 2003 8 191 209 10.1080/13546800244000292 16571560

[B33] Euteneuer F Schaefer F Stuermer R Boucsein W Timmermann L Barbe MT Ebersbach G Otto J Kessler J Kalbe E Dissociation of decision-making under ambiguity and decision-making under risk in patients with Parkinson's disease: a neuropsychological and psychophysiological study Neuropsychologia 2009 47 2882 2890 10.1016/j.neuropsychologia.2009.06.014 19545579

[B34] Monetta L Grindrod CM Pell MD Irony comprehension and theory of mind deficits in patients with Parkinson's disease Cortex 2009 45 972 981 10.1016/j.cortex.2009.02.021 19371867

[B35] Peron J Vicente S Leray E Drapier S Drapier D Cohen R Biseul I Rouaud T Le Jeune F Sauleau P Verin M Are dopaminergic pathways involved in theory of mind? A study in Parkinson's disease Neuropsychologia 2009 47 406 414 10.1016/j.neuropsychologia.2008.09.008 18845171

[B36] Bodden ME Mollenhauer B Trenkwalder C Cabanel N Eggert KM Unger MM Oertel WH Kessler J Dodel R Kalbe E Affective and cognitive Theory of Mind in patients with parkinson's disease Parkinsonism Relat Disord 2010 16 466 470 10.1016/j.parkreldis.2010.04.014 20538499

[B37] Peron J Le Jeune F Haegelen C Dondaine T Drapier D Sauleau P Reymann JM Drapier S Rouaud T Millet B Verin M Subthalamic nucleus stimulation affects theory of mind network: a PET study in Parkinson's disease PLoS One 2010 5 e9919 10.1371/journal.pone.0009919 20360963 PMC2847915

[B38] Roca M Torralva T Gleichgerrcht E Chade A Arevalo GG Gershanik O Manes F Impairments in social cognition in early medicated and unmedicated Parkinson disease Cogn Behav Neurol 2010 23 152 158 10.1097/WNN.0b013e3181e078de 20829664

[B39] Tsuruya N Kobayakawa M Kawamura M Is “reading mind in the eyes” impaired in Parkinson's disease? Parkinsonism Relat Disord 2011 17 246 248 10.1016/j.parkreldis.2010.09.001 20889365

[B40] Costa A Peppe A Martini M Coletta K Oliveri M Caltagirone C Carlesimo GA Parkinsonian patients with deficits in the dysexecutive spectrum are impaired on theory of mind tasks Behav Neurol 2012 Epub ahead of print 10.3233/BEN-129018 PMC5214465 23242360

[B41] Roca M Manes F Chade A Gleichgerrcht E Gershanik O Arevalo GG Torralva T Duncan J The relationship between executive functions and fluid intelligence in Parkinson's disease Psychol Med 2012 42 2445 2452 10.1017/S0033291712000451 22440401 PMC3466050

[B42] Santangelo G Vitale C Trojano L Errico D Amboni M Barbarulo AM Grossi D Barone P Neuropsychological correlates of theory of mind in patients with early Parkinson’s disease Mov Disord 2012 27 98 105 10.1002/mds.23949 21915910

[B43] Yu RL Wu RM Chiu MJ Tai CH Lin CH Hua MS Advanced Theory of Mind in patients at early stage of Parkinson's disease Parkinsonism Relat Disord 2012 18 21 24 10.1016/j.parkreldis.2011.08.003 21868278

[B44] Shamay-Tsoory SG Harari H Aharon-Peretz J Levkovitz Y The role of the orbitofrontal cortex in affective theory of mind deficits in criminal offenders with psychopathic tendencies Cortex 2010 46 668 677 10.1016/j.cortex.2009.04.008 19501818

[B45] Brothers L Ring B A neuroethological framework for the representation of minds J Cogn Neurosci 1992 4 107 118 10.1162/jocn.1992.4.2.107 23967887

[B46] Shamay-Tsoory SG Tomer R Berger BD Aharon-Peretz J Characterization of empathy deficits following prefrontal brain damage: the role of the right ventromedial prefrontal cortex J Cogn Neurosci 2003 15 324 337 10.1162/089892903321593063 12729486

[B47] Hynes CA Baird AA Grafton ST Differential role of the orbital frontal lobe in emotional versus cognitive perspective-taking Neuropsychologia 2006 44 374 383 10.1016/j.neuropsychologia.2005.06.011 16112148

[B48] Kalbe E Schlegel M Sack AT Nowak DA Dafotakis M Bangard C Brand M Shamay-Tsoory S Onur OA Kessler J Dissociating cognitive from affective theory of mind: a TMS study Cortex 2010 46 769 780 10.1016/j.cortex.2009.07.010 19709653

[B49] Montag C Schubert F Heinz A Gallinat J Prefrontal cortex glutamate correlates with mental perspective-taking PLoS One 2008 3 e3890 10.1371/journal.pone.0003890 19060949 PMC2586651

[B50] Zgaljardic DJ Borod JC Foldi NS Mattis PJ Gordon MF Feigin A Eidelberg D An examination of executive dysfunction associated with frontostriatal circuitry in Parkinson's disease J Clin Exp Neuropsychol 2006 28 1127 1144 10.1080/13803390500246910 16840240 PMC4456005

[B51] Kemp J Despres O Sellal F Dufour A Theory of Mind in normal ageing and neurodegenerative pathologies Ageing Res Rev 2012 11 199 219 10.1016/j.arr.2011.12.001 22186031

[B52] Fine C Lumsden J Blair RJ Dissociation between 'theory of mind' and executive functions in a patient with early left amygdala damage Brain 2001 124 287 298 10.1093/brain/124.2.287 11157556

[B53] Rowe AD Bullock PR Polkey CE Morris RG “Theory of mind” impairments and their relationship to executive functioning following frontal lobe excisions Brain 2001 124 600 616 10.1093/brain/124.3.600 11222459

[B54] Lough S Gregory C Hodges JR Dissociation of social cognition and executive function in frontal variant frontotemporal dementia Neurocase 2001 7 123 130 10.1093/neucas/7.2.123 11320160

[B55] Channon S Crawford S The effects of anterior lesions on performance on a story comprehension test: left anterior impairment on a theory of mind-type task Neuropsychologia 2000 38 1006 1017 10.1016/S0028-3932(99)00154-2 10775711

[B56] Stuss DT Gallup GG Jr Alexander MP The frontal lobes are necessary for ‘theory of mind’ Brain 2001 124 279 286 10.1093/brain/124.2.279 11157555

[B57] Carlson SM Moses LJ Breton C How specific is the relation between executive function and Theory of Mind? Contributions of inhibitory control and working memory Infant Child Dev 2002 11 73 92 10.1002/icd.298

[B58] Hoops S Nazem S Siderowf AD Duda JE Xie SX Stern MB Weintraub D Validity of the MoCA and MMSE in the detection of MCI and dementia in Parkinson disease Neurology 2009 73 1738 1745 10.1212/WNL.0b013e3181c34b47 19933974 PMC2788810

[B59] Troster AI A Precis of Recent Advances in the Neuropsychology of Mild Cognitive Impairment(s) in Parkinson’s Disease and a Proposal of Preliminary Research Criteria J Int Neuropsychol Soc 2011 17 393 406 10.1017/S1355617711000257 21473805

[B60] Litvan I Goldman JG Troster AI Schmand BA Weintraub D Petersen RC Mollenhauer B Adler CH Marder K Williams-Gray CH Diagnostic criteria for mild cognitive impairment in Parkinson's disease: Movement Disorder Society Task Force guidelines Mov Disord 2012 27 349 356 10.1002/mds.24893 22275317 PMC3641655

[B61] Modinos G Obiols JE Pousa E Vicens J Theory of Mind in different dementia profiles J Neuropsychiatry Clin Neurosci 2009 21 100 101 10.1176/appi.neuropsych.21.1.100 19359462

